# Novel Multi-Antigen Orf-Virus-Derived Vaccine Elicits Protective Anti-SARS-CoV-2 Response in Monovalent and Bivalent Formats

**DOI:** 10.3390/vaccines12050490

**Published:** 2024-05-01

**Authors:** Dominique Julien Burri, Louis Renz, Melanie Mueller, Felix Pagallies, Ute Klinkhardt, Ralf Amann, Madiha Derouazi

**Affiliations:** 1 Speransa Therapeutics, Frankfurt am Main, 60327 Frankfurt, Germany; d.burri@speransatherapeutics.com (D.J.B.); louis.r01001@gmail.com (L.R.); u.klinkhardt@speransatherapeutics.com (U.K.); 2 Institute of Immunology, University Hospital of Tübingen, 72016 Tübingen, Germany; melanie.mueller@uni-tuebingen.de (M.M.);

**Keywords:** Orf virus, COVID-19 vaccine, parapoxvirus

## Abstract

Prime-2-CoV_Beta is a novel Orf virus (ORFV)-based COVID-19 vaccine candidate expressing both the nucleocapsid and spike proteins of SARS-CoV-2 with the receptor-binding domain (RBD) of the Beta strain. This candidate was shown to be safe and immunogenic in a first-in-human Phase I clinical trial. With the shift in the immune landscape toward the Omicron variant and the widespread vaccine- and/or infection-derived immunity, further pre-clinical research was needed to characterize Prime-2-CoV. Here, we quantified the humoral and cellular response to Prime-2-CoV_Beta in pre-immunized mice and compared the protective efficacy of mono- and bivalent variant-based Prime-2-CoV vaccine candidates in hamsters. Prime-2-CoV_Beta induced robust humoral and cellular immune responses in naïve animals but did not further boost antibody titers in the tested setting when given as repeat booster at short interval. We furthermore showed that Prime-2-CoV_Beta-based mono- and bivalent immunization strategies produced comparable immunogenicity and protection from infection. Our results highlight the potential of the Orf virus as a vaccine platform against SARS-CoV-2 and potentially other infectious viruses.

## 1. Introduction

The COVID-19 pandemic, caused by severe acute respiratory syndrome coronavirus 2 (SARS-CoV-2), underscored the need for new and innovative vaccine platforms. One such platform, based on the highly attenuated D1701-VrV strain of the Orf virus (ORFV) [1]—a parapoxvirus, which naturally infects goats and sheep—has demonstrated several advantages over conventional vector-based platforms, including a good safety profile, the ability to induce potent immunogenicity against recombinant antigens, and more importantly, the absence of a durable immunity against the vector itself [2,3,4].

To leverage these advantages, we generated and investigated a D1701-VrV-derived multi-antigen SARS-CoV-2 vaccine candidate named Prime-2-CoV, expressing the full-length spike (S) protein and the nucleocapsid (N) protein of the original Wuhan strain of SARS-CoV-2. In response to the evolving landscape of SARS-CoV-2 variants, Prime-2-CoV was updated to express the receptor-binding domain (RBD) of the Beta (B.1.351) and Delta (B.1.617.2) strains. Prime-2-CoV_Beta was shown to be safe and immunogenic in a first-in-human Phase I clinical trial study (ORFEUS, NCT05367843, manuscript under review). In response to the rise of the Omicron strain, Prime-2-CoV was re-designed to express the full spike and nucleocapsid protein sequences of Omicron (BA.1): Prime-2-CoV_Omicron (BA.1).

Vaccine candidates based on the ancestral D614G, Beta and Delta strains were tested extensively in mouse, hamster and non-human primate models in which they elicited a good immune response [5]. They also protected hamsters against severe disease when challenged with the SARS-CoV-2 ancestral D614 strain [5]. With the success of the worldwide COVID-19 vaccination campaign and the wide spread of the Omicron strain, the immune landscape of the population had drastically changed, with SARS-CoV-2 antibody seroprevalence reaching more than 90% of the population in Europe [6]. Therefore, additional pre-clinical investigations were conducted to characterize the impact of pre-existing immunity on the magnitude of the immune response to Prime-2-CoV_Beta. Studies have shown that heterologous COVID-19 vaccine regimens lead to neutralizing antibody titers at least comparable or higher than homologous vaccination regimens [7]. Therefore, we evaluated whether this would apply to Prime-2-CoV as well.

In light of recommendations for Omicron-adapted bivalent mRNA vaccines to address the reduced effectiveness of SARS-CoV-2 monovalent vaccines against the Omicron strain [8], we tested this strategy by designing bivalent formulations containing either Delta-Omicron (BA.1) or Beta-Omicron (BA.1) Prime-2-CoV vaccine candidates in equal amounts.

Our study evaluated the humoral and cellular response to Prime-2-CoV_Beta in pre-immunized mice and compared the antibody induction and protective efficacy of mono- and bivalent variant-specific Prime-2-CoV vaccine candidates in hamsters. We showed that Prime-2-CoV_Beta administered as mono- or bivalent vaccine formulation in hamsters resulted in a significant reduction in viral loads in the lungs, indicating a protective effect against homo- and heterologous SARS-CoV-2 infections. This highlights the potential of the ORFV-based vaccine platform for future vaccine development.

## 2. Materials and Methods

### 2.1. Vaccine Production

Prime-2-CoV_Beta batch SPEk103 was manufactured and vialed by ABL Europe (Illkirch-Graffenstaden, France) and Nova Laboratories (Wigston, UK) according to good manufacturing practices. In brief, Prime-2-CoV_Beta was amplified in HEK293 cells, followed by a downstream process comprising several membrane-based filtration steps, nuclease treatment for breakdown of contaminating DNA and two chromatographic purification steps. The vaccine was formulated with recombinant human Albumin in vials and stored at −20 °C until use.

For the UV inactivation of Prime-2-CoV_Beta, vials of Prime-2-CoV_Beta SPEk103 were thawed on ice and distributed in aliquots of 50 μL in 1.5 mL Eppendorf tubes. The tubes were left open on ice, and the rack was placed 30 cm away from the UV light in a biosafety cabinet. The infectivity of the virus treated with UV for 5, 10, 15, 30 or 45 min irradiation time was assessed using the plaque assay and immunocytometry assay. It was determined that 15 min irradiation time was the minimum time resulting in complete inactivation of the virus. However, to attain a comfortable safety margin, we chose a 30 min irradiation time for all further experiments.

For the hamster challenge study, vaccine batches of Prime-2-CoV_Beta and Omicron (BA.1) were manufactured by Prime Vector Technology (Tübingen, Germany) in HEK293 cells and purified/concentrated on sucrose cushion via ultracentrifugation at 49,280× *g* for 30 min. The purified vaccine was frozen at −20 °C until use.

### 2.2. Humoral Response against SARS-CoV-2 Beta RBD

RBD strain-specific antibody endpoint titers were measured via enzyme-linked immunosorbent assay (ELISA). Briefly, MaxiSorp™ 96-well plates (Thermo Fisher Scientific, Waltham, MA, USA, 430341) were coated with 1 μg/mL of purified RBD recombinant protein (SinoBiological, Wayne, PA, USA, 40592-V08H85 (Beta)) diluted in PBS overnight at 4 °C. After three washes, the plates were blocked for 2 h in 200 μL/well of blocking buffer PBS-T (PBS, Tween20 0.05%) and 1% bovine serum albumin (BSA) (PAN-Biotech, Aidenbach, Germany, P06-1391100) at 37 °C. The plates were subsequently washed with PBS-T. Then, serial dilutions of hamster serum, diluted in PBS-T (50 μL), were added to the wells and incubated for 2 h at 37 °C. After three washes with PBS-T, HRP-linked anti-hamster antibody (Bethyl Laboratories, Montgomery, TX, USA, A140-101P) was added (1:10,000) and incubated for 1 h at 37 °C. The plates were then washed five times with 200 μL/well of PBS-T and 50 μL/well of TMB substrate (BioLegend, San Diego, CA, USA, 421101) added for 5 min in the dark. The reaction was quenched with 50 μL of Stop solution (H_2_SO_4_ 0.18 M), and well absorbance at 450 nm was immediately read on a Varioskan™ Lux microplate reader (Thermo Fisher Scientific, VL0000D0). Absorbance data were analyzed using Graphpad Prism (10.1.1) and plotted against dilution factors, and a four-parameter fit curve was applied. The cut-off was calculated as the mean of the blank plus four times the SD of the blank.

### 2.3. Humoral Response against Orf Virus Protein F1L (ORFV059)

Anti-F1L antibody endpoint titers were measured via ELISA, as previously described, with modifications [9]. Briefly, MaxiSorp™ 96-well plates (Thermo Fisher Scientific, 430341) were coated with 1 μg/mL of purified F1L recombinant protein (Genscript, Piscataway, NJ, USA, custom production based on ORFV D1701 PP83 (ORFV059), residue 1-289) in 0.05 M carbonate buffer (pH9.0) overnight at 4 °C. After three washes with phosphate-buffered saline (PBS), the plates were blocked for 2 h in 200 μL/well of blocking buffer PBS-T (PBS, Tween20 0.05%) and 1% bovine serum albumin (BSA) (PAN-Biotech, P06-1391100) at 37 °C. The plates were subsequently washed three times with 300 μL/well of PBS-T, and serial dilutions of mouse serum in PBS-T (50 μL) were added to the wells and incubated for 2 h at 37 °C. After three washes with PBS-T, HRP-conjugated anti-mouse IgG-Fc fragment secondary antibody (Bertyl Laboratories, A90-131P) was incubated for 1 h at 37 °C. The plates were then washed five times with 200 μL/well of PBS-T and 50 μL/well of TMB substrate (BioLegend, 421101) added for 5 min in the dark. The reaction was quenched with 50 μL of Stop solution (H_2_SO_4_ 0.18 M), and well absorbance at 450 nm was immediately read on a Varioskan™ Lux microplate reader (Thermo Fisher Scientific, VL0000D0). The cut-off was calculated as the mean of the blank plus four times the SD of the blank.

### 2.4. Detection and Quantification of Strain-Specific Neutralizing Antibodies

To detect and quantify SARS-CoV-2 strain-specific neutralizing antibodies in hamster sera, a surrogate virus neutralization test (sVNT) was performed using the cPass SARS-CoV-2 Neutralization Antibody Detection Kit (Genscript, L00847-C). The assay was performed according to the manufacturer’s protocol. Samples of the following were diluted 1:10 with the provided dilution buffer and added to the pre-diluted strain-specific RBD-HRP: wild-type (Genscript, Z03594), Beta (Genscript, Z03596), Delta B.1.617.2 (Genscript, Z03614), Omicron BA.1 (B1.1.529) (Genscript, Z03730). Mixed samples were incubated for 30 min at 37 °C. Then, 100 μL was added to the provided ACE2 pre-coated plates and incubated for 15 min at 37 °C. The SARS-CoV-2 Neutralizing Antibody Standard (Genscript, A02087) was used as calibrator control, as recommended in the manufacturer’s instructions. All results were calculated as percentage of binding inhibition vs. negative controls.

### 2.5. Flow Cytometry Analysis of Prime-2-CoV_Beta-Stimulated Human PBMCs

Human PBMCs collected from likely COVID-19-vaccinated donors between February 2022 and February 2023 were isolated from buffy coats by means of Ficoll density gradient centrifugation and infected for 24 h with 5 MOI of Prime-2-CoV_Beta (SPEk103) or left untreated. Cells were collected by scraping, washed in PBS and incubated for 30 min at 4 °C with the following antibody cocktail in PBS: anti-CD14 APC (BD Biosciences, San Jose, CA, USA, 561383), anti-CD4 BV605 (BD Biosciences, 562843), anti-CD8 APC-H7 (BD Biosciences, 561423), anti-CD19 BV786 (BD Biosciences, 563325), anti-CD3 PE-Cy7 (BD Biosciences, 557749), anti-CD56 PE (BD Biosciences, 556647), anti-CD25 BV421 (BD Biosciences, 567485), LIVE/DEAD™ Fixable Aqua stain (Invitrogen, Waltham, MA, USA, L34957). After three washes in PBS, cells were fixed in 4% formaldehyde for 20 min, washed and finally resuspended in FACS buffer. Data were acquired with an Attune NxT cytometer (Thermo Fisher Scientific). Data analysis was performed using Kaluza software (version 2.1, Beckman Coulter, Brea, CA, USA).

### 2.6. Luminex Assay

Human PBMC medium was collected at the timepoints indicated in the text, centrifuged to remove debris and stored at −20 °C. The cytokine and chemokine contents of the media were measured using an Inflammation 20-Plex Human ProcartaPlex™ Panel Luminex assay (Invitrogen, EPX200-12185-901) according to the manufacturer’s instructions. Analysis was performed using Luminex software (Thermo Fisher Scientific). Fold changes were calculated after averaging the replicates.

### 2.7. Intracellular Cytokine Staining (ICS) Assay

Mouse spleens were harvested and shipped fresh in wash medium (RPMI-1640 (Gibco, Thermo Fisher, 61870036), 10% inactivated FCS and 1% penicillin/streptomycin) at 4 °C. Splenocytes were isolated by crushing the spleens through a 70 μm cell strainer in wash medium. After Lymphoprep™ isolation (Serumwerk, Bernburg, Germany, 1114545), splenocytes were washed in wash medium and finally resuspended in CTL medium (DMEM Glutamax, Thermo Fisher, 6% inactivated FCS, 10 mM HEPES, 10 μM Beta-Mercaptoethanol, 1X mix aa (L-Arg, L-Gln, L-Asn), 1% P/S) at a density of 2 × 10^7^ cells/mL. The SARS-CoV-2 spike protein stimulation peptide pool (custom synthesis, 15 mer, 10 aa overlap based on YP_009724390.1, Peptides&Elephants, Hennigsdorf, Germany) was diluted to 2 g/mL in CTL medium together with anti-CD28 (BD Biosciences, 553294), anti-CD49d (BD Biosciences, 553314) co-stimulatory antibodies and anti-CD107 (BD Biosciences, 564348) degranulation marker. Cell stimulating cocktail (Invitrogen, 00-4970-03) was used as positive control. Cells (2 × 10^6^ cells/well) were incubated with SARS-CoV-2 spike protein stimulation peptide pool or controls for 1 h at 37 °C, at which point the Golgi Plug™ solution was added according to the manufacturer’s instructions (BD Biosciences, 555029). The plates were incubated for 5 h at 37 °C and then placed at 4 °C until staining. For staining, cells were transferred in V-bottom plates, centrifuged for 2 min at 751 rcf and resuspended in 15 μL of Fc Block solution containing CD16/CD32 Mouse Fc Block (BD Biosciences, 553142) at 0.5 μg/10^6^ cells. After 10 min of incubation on ice, extracellular staining solution was added and incubated for 20 min at 4 °C. The extracellular staining solution consisted of anti-CD3 PerCP Cy5.5 (BD Biosciences, 560527), anti-CD11b PECy7 (BD Biosciences, 561098), anti-CD45 BV421 (BD Biosciences, 563890), anti-CD8 APC-H7 (BD Biosciences, 560182), anti-CD4 BV-480 (BD Biosciences, 565634) and LIVE/DEAD™ Fixable Yellow stain (Invitrogen, L34959) in FACS buffer. Cells were then washed twice in FACS buffer, resuspended in Cytofix/Cytoperm buffer (BD Biosciences, 555028) for 20 min at 4 °C, washed twice in Cytofix/Cytoperm buffer and incubated with anti-TNF PE (BD Biosciences, 561063), anti-IFN-γ APC (BD Biosciences, 562018) and anti-IL-2 Alexa488 (BD Biosciences, 557725) for 30 min at 4 °C. After three washes in Cytofix/Cytoperm buffer, cells were resuspended in FACS buffer, and data were acquired with an Attune NxT cytometer (Thermo Fisher Scientific). Data analysis was performed using Kaluza software (version 2.1, Beckman Coulter). The signal in the negative control (DMSO) wells was subtracted from the signal in the spike-pool-stimulated wells.

### 2.8. Animal Experimentation

#### 2.8.1. Mouse Experimentation

##### Ethical Statement

Animal experimentations were conducted by Oncodesign Services (Villebon-sur-Yvette, France). Animal housing and experimental procedures were conducted according to the French and European Regulations and the National Research Council Guide for the Care and Use of Laboratory Animals. The animal facility is recognized by French authorities (CFH: Agreement N° B 91 962 106). All animal procedures (including surgery, anesthesia and euthanasia, as applicable) used in the current study (220462/ethical protocol: 2022-09) were submitted to the Institutional Animal Care and Use Committee of Oncodesign Services (Oncomet) approved by French authorities [CNREEA agreement N° 91 (Oncodesign Services)].

##### Effect of Anti-Spike Pre-Existing Immunity on Response to Prime-2-CoV Vaccination

A total of fifteen healthy female CD1 mice, 8–12 weeks old (Charles River), were randomized based on individual body weight into three groups of five animals each. On days 0 and 21, mice were injected IM in the upper thigh, after application of a topical local analgesic, with 50 μL of a previously approved monovalent mRNA vaccine expressing the ancestral D614G spike protein (5 μg) or with a PBS control. On days 35 and 56, mice were injected IM in the upper thigh with 50 μL of Prime-2-CoV_Beta (SPEj102, 2.05 × 10^6^ PFU) diluted in PBS or PBS control. Animals were monitored after the injections and throughout the duration of the study. On sampling days 0, 21, 35, 56 and 70, blood (approximately 80 μL) was collected (before vaccination when applicable) via jugular vein puncture into collection tubes with a clot activator and allowed to coagulate for 30 min. The tubes were then centrifuged (2000× *g*, 10 min, RT), and the serum was transferred to clean tubes and frozen at −80 °C. On day 70, spleens collected post-mortem for splenocyte isolation and ICS were handled as described in Section 2.7 of the Materials and Methods section.

#### 2.8.2. Hamster Experimentation

##### Ethical Statement

Male Syrian golden hamsters, 9–10 weeks old, were obtained from Janvier Labs (Le Genest-Saint-Isle, France). The animals were housed and all procedures were performed by Viroclinics at their pre-clinical facility in Schaijk (The Netherlands) under conditions meeting the standard of Dutch law for animal experimentation and in accordance with Directive 2010/63/EU of the European Parliament and of the Council from 22 September 2010 on the protection of animals used for scientific purposes. Ethical approval for the experiment was registered under protocol number AVD27700202114492-WP46.

##### Immunization

Animals were randomly assigned to an experimental group according to the agreed study design, with five animals per group, upon arrival at the facility. Using a prime–boost regimen, animals (six groups of five 9–10-week-old male *Mesocricetus auratus* per group for each challenge condition, sixty animals in total) were immunized 4 weeks apart via the intramuscular route of administration (IM) using a syringe fitted with a 29 G (0.33 × 12.7 mm) needle. Vaccine vials were thawed at room temperature, and 0.1 mL (containing 3 × 10^7^ plaque-forming units (PFUs) of Prime-2-CoV variants in monovalent formulation, or 2 × 1.5 × 10^7^ PFU of each variant tested in bivalent formulation) was injected into the outer thigh (biceps femoris). The control animals received 0.1 mL of PBS. The inoculum was administered to the same limb for both the prime and boost immunizations.

##### Challenge

Four weeks following the booster immunization, the animals were challenged by means of infection via the intranasal route of administration (IN) with either a 10^4^ TCID_50_/dose of SARS-CoV-2 Delta variant strain (VC-210180046) or a 10^4^ TCID_50_/dose of SARS-CoV-2 Omicron BA.1 variant strain (EMC_07/04/2021) in a total dose volume of 0.1 mL. Animals were held on their back, and the inoculum was administered equally over both nostrils using a pipette. Animals were then followed for four days post-challenge (p.c.) and euthanized by means of abdominal exsanguination under isoflurane anesthesia.

##### Sampling Post-Challenge

Prior to challenge on day 0 p.c. and days 1–4 p.c., a throat swab was collected and frozen at −80 °C for virological analyses. At the time of euthanasia, half of the lungs and nasal turbinate tissues were collected and frozen at −80 °C for virological analyses. The other half of the lungs and nasal turbinate, as well as trachea tissues, were fixed in 4% buffered formalin (Chemie Vertrieb GmbH & Co. Hannover KG, Hannover, Germany) for histopathology.

##### Viral Load in the Respiratory Tract

Detectable levels of replication-competent virus in throat swabs, lung and nasal turbinate tissues p.c. were analyzed at Viroclinics Schaijk (The Netherlands). Quadruplicate, 10-fold serial dilutions of samples were transferred to 96-well plates with Vero/TMPRSS2 cell culture monolayers and incubated for 1 h at 37 °C. Cell monolayers were washed prior to the addition of infection medium and incubation for 5 days at 37 °C. The presence of the cytopathic effect (CPE) was determined using the cell vitality marker WST-8. A WST-8 stock solution was prepared containing 4 µL WST-8 (Sigma Aldrich catalog # 96992) and 16 µL infection medium (1:5 dilution). To each well, 20 µL of the solution was added, and the plates were incubated for approximately 3 h at room temperature. Subsequently, the plates were measured for optical density at 450 nm (OD450). Viral titers (Log_10_ TCID_50_/mL or /g) were calculated using the Spearman–Karber method. The lower limit of detection (LLOD) for titration of throat swab samples was 0.8 Log_10_ TCID_50_/mL. Titers were classified as undetectable when <0.8 Log_10_ TCID_50_/mL or detectable when ≥0.8 Log_10_ TCID_50_/mL. The LLOD for titration of tissue samples was variable, as it was dependent on the weight of the tissue sample. Sample titers were classified as undetectable when <[value] Log_10_ TCID_50_/g or detectable when ≥[value] Log_10_ TCID_50_/g for the individual sample.

Detectable levels of viral RNA present in throat swabs, lung and nasal turbinate tissues p.c. were analyzed at Viroclinics Schaijk (The Netherlands). The extraction and purification of RNA was performed using the Roche MagNA Pure 96 system. Prior to extraction, each test and negative control sample was spiked with phocine distemper virus (PDV) as an internal control for the step. Reverse transcription and qPCR were performed using an Applied Bio 7500 Real-Time PCR System and Applied Biosystems TaqMan Sarbeco E gene expression assay, with the following beta coronavirus E-specific primers and probe: E_Sarbeco_F1, 5′–ACAGGTACGTTAATAGTTAATAGCGT–3′; E_Sarbeco_R2, 5′ ATATTGCAGCAGTACGCACACA–3′; E_Sarbeco_P1, 5′–FAM-ACACTAGCCATCCTTACTGCGCTTCG-BHQ1-3.

The number of copies (Log_10_ CP/mL/g) in the different samples was calculated against a trended (four-point) positive virus control included in each of the assays. The LLOD for PCR of throat swab samples was 1.9 Log_10_ CP/mL. The results were classified as undetectable when <1.9 Log_10_ CP/mL or detectable when ≥1.9 Log_10_ CP/mL. The LLOD for PCR of tissue samples was variable, as it was dependent on the weight of the tissue sample. The results were classified as undetectable when <[value] Log_10_ CP/g or detectable when ≥[value] Log_10_ CP/g for the individual sample.

##### Histopathology

Histopathological analysis was performed on lung, nasal turbinate and trachea tissues sampled on day 4 p.c. Formalin-fixed, paraffin-embedded samples were cut into 2 μm thick serial sections and stained with hematoxylin and eosin (H&E). Sections of the nasal turbinate, trachea and lung were scanned using an Olympus VS200 digital slide scanner (Olympus Deutschland GmbH, Hamburg, Germany) and evaluated in a blinded manner using a semi-quantitative scoring system with emphasis on inflammation, degeneration and regeneration. Scoring was carried out as follows. Alveolitis severity, bronchitis/bronchiolitis severity, rhinitis severity, tracheitis severity: 0 = no inflammatory cells, 1 = few inflammatory cells, 2 = moderate number of inflammatory cells, 3 = many inflammatory cells. Alveolitis extent: 0 = 0%, 1 = <25%, 2 = 25–50%, 3 = >50%. Alveolar oedema presence, alveolar hemorrhage presence, type II pneumocyte hyperplasia presence: 0 = no, 1 = yes. Extent of peribronchial/perivascular cuffing: 0 = none, 1 = 1–2 cells thick, 2 = 3–10 cells thick, 3 = >10 cells thick.

### 2.9. Statistical Analysis

Statistical analysis was conducted using GraphPad Prism (v10.1.1). Comparisons between two groups or two conditions were assessed using unpaired Student’s *t*-test. For comparisons involving more than two groups and timepoints, two-way ANOVA was used. Symbols of statistical significance: *, *p* < 0.05; **, *p* < 0.01; ***, *p* < 0.001; ****, *p* < 0.0001.

## 3. Results

### 3.1. Prime-2-CoV

Prime-2-CoV is a novel Orf virus (ORFV)-based vaccine against SARS-CoV-2. Codon-optimized spike and nucleocapsid DNA sequences from the SARS-CoV-2 ancestral strain D614 were introduced via homologous recombination into Orf virus strain D1701-VrV at loci, which had previously demonstrated efficient transgene expression [1,2]. The spike coding sequence also contained mutations (AA682-685 and K986P, V987P) known for their ability to stabilize the protein in its pre-fusion conformation [10,11,12]. In response to the emergence of new SARS-CoV-2 variants, Prime-2-CoV was updated with the receptor-binding domain (RBD) of the Beta (B.1.351) and Delta (B.1.617.2) strains.

**Figure 1:**
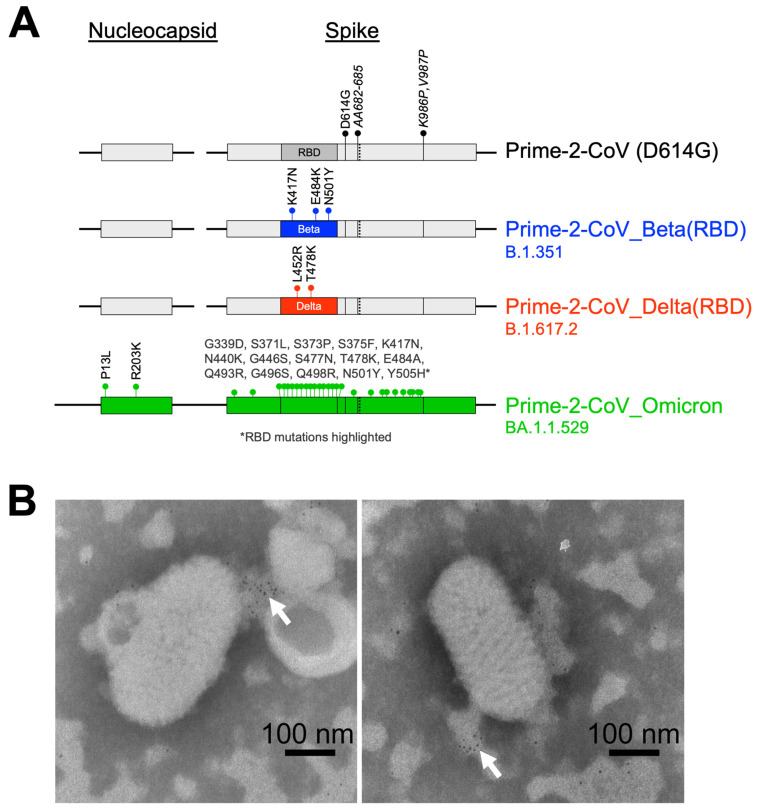
(**A**) Schematic representation of the Prime-2-CoV vaccine candidates used in this study, * RBD mutations highlighted; (**B**) Electron microscopy images of gold-labeled anti-SARS-CoV-2 spike protein detection in Prime-2-CoV_Beta preparation. White arrows highlight the locations of concentrated labeling.

The replacement of ancestral-strain-based variants in late 2021 by the Omicron (BA.1) strain, with its significant genetic divergence, required a substantial overhaul of Prime-2-CoV. A new vaccine candidate Prime-2-CoV_Omicron (BA.1.1.529) was engineered, incorporating the full-length Omicron spike (engineered with the pre-fusion stabilizing mutations and the removal of the furin cleavage site) and nucleocapsid protein sequences (Figure 1A). Electron microscopy analysis revealed that Prime-2-CoV_Beta has the characteristic shape of *parapoxviruses* and does not display SARS-CoV-2 spike protein on its surface, with gold-labeled anti-spike antibodies concentrating on cell membrane debris but not on the Prime-2-CoV_Beta virus itself (Figure 1B).

### 3.2. Prime-2-CoV_Beta Infection of Human PBMC

**Figure 2:**
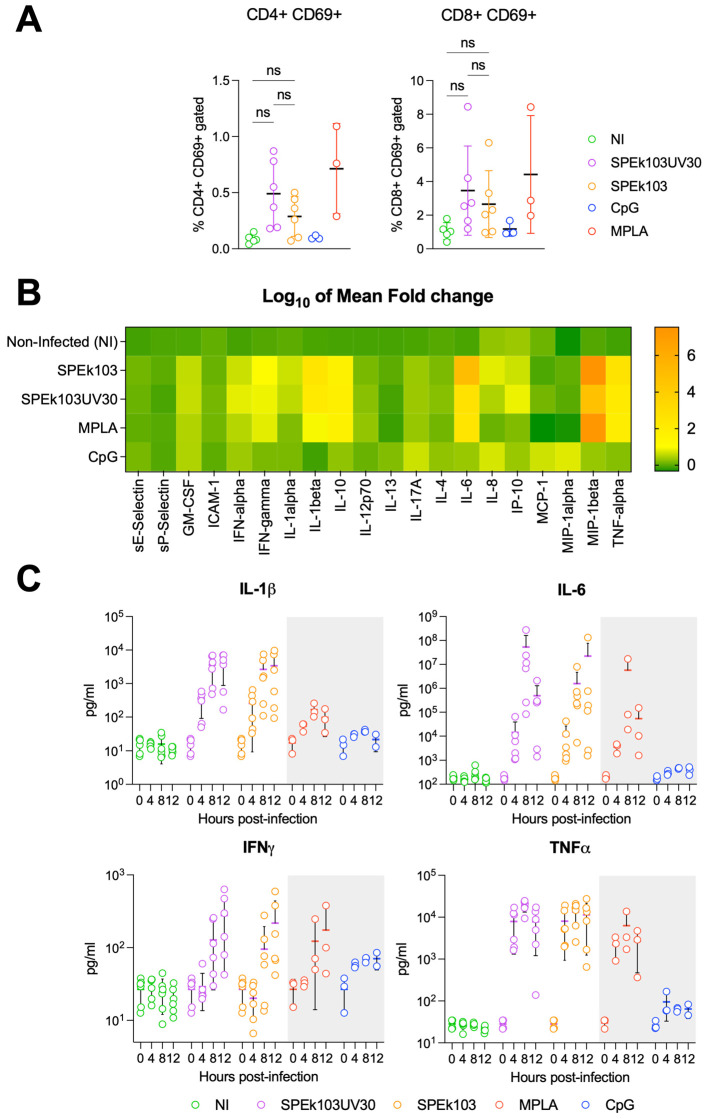
(**A**) Percentage of activated (CD69+) CD4+ (left panel) and CD8+ T cells (right panel) after 24 h infection of human PBMC with vaccine candidates Prime-2-CoV_Beta (SPEk103), UV-inactivated Prime-2-CoV_Beta (SPEk103UV30), CpG or MPLA controls; (**B**) Heat map representation of the Log_10_ of the mean fold changes between time 0 and 12 h post-infection of human PBMC measured via Luminex assay; (**C**) Cytokine secretion profiles in pg/mL at 0, 4, 8 and 12 h post-infection of human PBMC. IL-1β, IL-6, IFNγ and TNFα are depicted. Statistical analysis was conducted using GraphPad Prism (v10.1.1). Comparisons between two groups were assessed using unpaired Student’s *t*-test. ns: Non-significant.

The effects of D1701-VrV (the Prime-2-CoV_Beta parental strain) infection of human peripheral blood mononuclear cells (PBMCs) were previously investigated [13]. In this study, the Orf virus (ORFV) was found to preferentially target professional antigen-presenting cells (APCs) and enter APCs primarily through macropinocytosis. ORFV-infected APCs displayed an activated phenotype, essential for APC-mediated lymphocyte activation. However, the effector cytokine secretion results of Prime-2-CoV_Beta infection of PBMC were lacking. Therefore, we infected human PBMCs originating from likely COVID-19-vaccinated donors with Prime-2-CoV_Beta (batch SPEk103) or UV-inactivated Prime-2-CoV_Beta (SPEk103UV30). A distinct feature of poxviruses is their ability to replicate in the cytoplasm of the cells, as they do not rely on the host cell’s nuclear machinery [14]. Consequently, the expression of transgenes, and thus, the host´s immune response against the transgenes, is dependent on the integrity of the viral DNA, as no transgene protein is present on the surface of the viral particle. This feature enables the control of transgene expression in poxvirus vectors, such as Prime-2-CoV_Beta. Exposure to ultraviolet (UV) light for 30 min completely abrogates the expression of spike transgene 24 and 48 h post-infection (Supplementary Figure S1B,C). In contrast, expressions of the transgenes’ SARS-CoV-2 spike, nucleocapsid and ORFV control protein F1L (ORFV059) in non-UV-treated Prime-2-CoV_Beta were detected 4 h–8 h post-infection by Western blot (Supplementary Figure S1A). Flow cytometry analysis of human PBMCs infected for 24 h with either Prime-2-CoV_Beta or its UV-inactivated form revealed similar activation of CD4+ and CD8+ T cells, regardless of the expression of the transgenes (Figure 2A), hinting at a mechanism of T-cell activation independent of the transgene expression.

It was previously shown that the Orf virus predominantly targets antigen-presenting cells in vitro and activates the STING pathway [13]. We further characterized the T-cell activation and effector cytokines’ release of PBMC via cytokine profiling at diverse timepoints ranging from 0 to 12 h post-Prime-2-CoV_Beta infection. Both Prime-2-CoV_Beta and its UV-inactivated version triggered a cytokine expression pattern similar to the monophosphoryl lipid A (MPLA) control 12 h after infection, with a clear induction of the pro-inflammatory cytokines IL-1β (2.46 Log_10_ mean fold change), IL-6 (5 Log_10_ mean fold change), IFNγ (1.02 Log_10_ mean fold change) and TNFα (2.59 Log_10_ mean fold change), as well as the macrophage inflammatory protein (MIP) Beta (7.54 Log_10_ mean fold change) (Figure 2B). UV inactivation of Prime-2-CoV_Beta did not significantly alter the cytokine expression profile of infected PBMCs, indicating that the adjuvant effect of Prime-2-CoV_Beta is independent of viral or transgene protein expression.

A detailed temporal analysis of the pro-inflammatory cytokines secreted by human PBMCs showed an increased concentration of IL-1β, IL-6 and TNFα starting at 4 h post-infection, with IFNγ amounts rising at 8 h post-infection (Figure 2C). No difference could be observed between the secretion timelines of Prime-2-CoV_Beta and its UV-inactivated form, both of which followed the same pattern as the MPLA control.

### 3.3. Effect of Pre-Existing Immunity on Prime-2-CoV_Beta Immunogenicity in Mice

**Figure 3:**
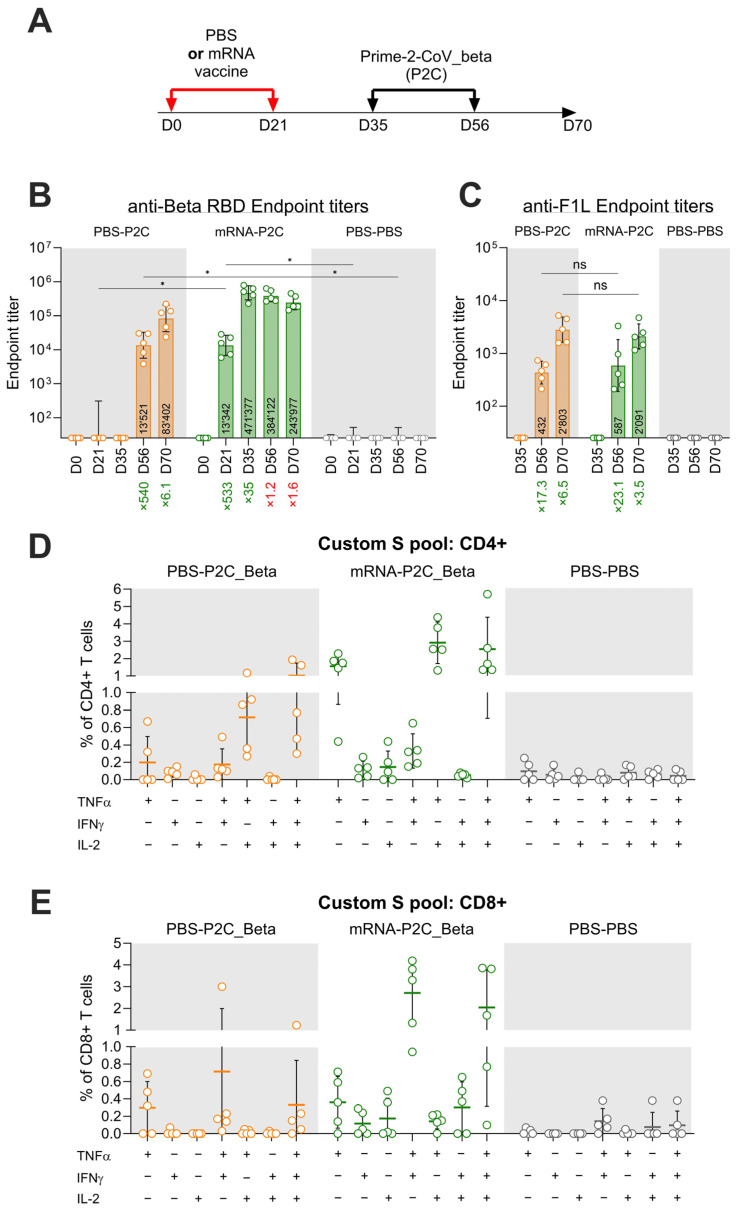
(**A**) Timeline overview of the study, with injections of either PBS or mRNA vaccine at days 0 and 21, followed by two vaccinations with Prime-2-CoV_beta at days 35 and 56; (**B**) Anti-SARS-CoV-2 Beta RBD endpoint titer measurement in mouse sera at indicated timepoints via ELISA; (**C**) Anti-F1L endpoint titers measured via ELISA. Numbers denote fold increase (green). Numbers within bars indicate the geometric mean for the bar. Error bars show the geometric SD; (**D**,**E**) Intracellular cytokine staining assay (ICS) of splenocytes isolated at day 70, showing CD4+ (**D**) and CD8+ I T-cell cytokine response to stimulation by a spike protein peptide pool. Statistical analysis was conducted using GraphPad Prism (v10.1.1). Comparisons were performed using two-way ANOVAs. Symbols of statistical significance: *, *p* < 0.05. Horizontal bars denote the mean, and error bars denote the standard deviation for the sample.

To mimic the current epidemiological situation characterized by a high prevalence of SARS-CoV-2 antibodies in the population, we evaluated the immune response elicited by Prime-2-CoV_Beta vaccination in pre-vaccinated mice. Initially, CD1 mice were vaccinated with 1/10th of the human dose of an approved monovalent mRNA vaccine (expressing ancestral D614G spike protein) or administered a mock vaccination with PBS at day 0 and day 21. Following this prime–boost immunization with spike mRNA or PBS control, all animals were subsequently vaccinated twice with Prime-2-CoV_Beta (at days 35 and 56) (Figure 3A). A robust induction of anti-Beta RBD antibody response was observed after the first injections with both mRNA and Prime-2-CoV_Beta vaccines, with a similar increase in the endpoint titers—533x (d56) and 540x (d21), respectively—compared to d0 (Figure 3B). Similarly, the first of the booster vaccinations resulted in increases in anti-Beta RBD endpoint titers for both mRNA (35-fold) and Prime-2-CoV_Beta (6.1-fold) conditions. However, subsequent Prime-2-CoV_Beta vaccinations in animals pre-vaccinated with mRNA vaccine resulted in a decrease in endpoint titers of 1.2x (d56) after the first and 1.6x (d70) after the second Prime-2-CoV_Beta injection.

As expected, the humoral immune response to the Orf virus protein F1L (ORF059), a surface antigen of Prime-2-CoV_Beta, was not impacted by prior immunization with the spike mRNA vaccine. The antibody endpoint titers against F1L increased by 17.3-fold in naïve animals and 23.1-fold in mRNA-pre-vaccinated animals. The anti-F1L response against the second Prime-2-CoV_Beta injection was also not significantly different between naïve (6.5-fold increase in titer) and mRNA pre-immune (3.5-fold increase in titer) animals. This indicates that pre-immunization with a spike mRNA vaccine does not adversely affect the humoral immune response against the Orf virus.

The cellular immune response elicited by Prime-2-CoV_Beta vaccination of naïve mice (PBS-P2C_Beta) or mice previously vaccinated with mRNA (mRNA-P2C_Beta) was assessed via intracellular cytokine staining (ICS) of splenocytes freshly isolated at day 70. In PBS-P2C_Beta, stimulation with a SARS-CoV-2 spike peptide pool resulted in 1% of multi-functional CD4+ T cells co-expressing TNFα, IFNγ and IL-2; 0.71% of CD4+ T cells co-expressing TNFα and IFNγ; and a small part expressing TNFα alone (0.19%) or TNFα and IFNγ (0.17%). These results were higher in mRNA-P2C_Beta, with 2.5% of multi-functional CD4+ T cells co-expressing the three cytokines; 2.9% expressing only TNFα and IL-2; and 1.5% of all CD4+ T cells expressing TNFα only (Figure 3D). Measurement of CD8+ T-cell frequencies in splenocytes of PBS-P2C_Beta revealed that 0.33% of CD8+ T cells were expressing TNFα, IFNγ and IL-2; 0.71% were co-expressing TNFα and IFNγ; and 0.29% were expressing TNFα only. In mRNA-P2C_Beta animals, 2% of CD8+ T cells were found to be expressing TNFα, IFNγ and IL-2; 2.7% were found to be co-expressing both TNFα and IFNγ; and 0.36% were found to be expressing only TNFα. In animals vaccinated with mRNA-P2C_Beta, 0.3% of CD8+ T cells present in splenocytes were co-expressing IFNγ and IL-2, but this population was not detected in naïve P2C-P2C animals (Figure 3E). When compared, none of the groups showed differences reaching statistical significance. As expected, no cytokine response was detected in CD4+ and CD8+ T cells from animals vaccinated with PBS. IL-4 was not detected in previous studies, including during Phase I clinical trial ORFEUS (data not shown), which is the reason why this cytokine was not assessed here.

### 3.4. Prime-2-CoV_Beta Protects Hamsters against Severe Disease as Effectively as Monovalent and Bivalent Injections

**Figure 4:**
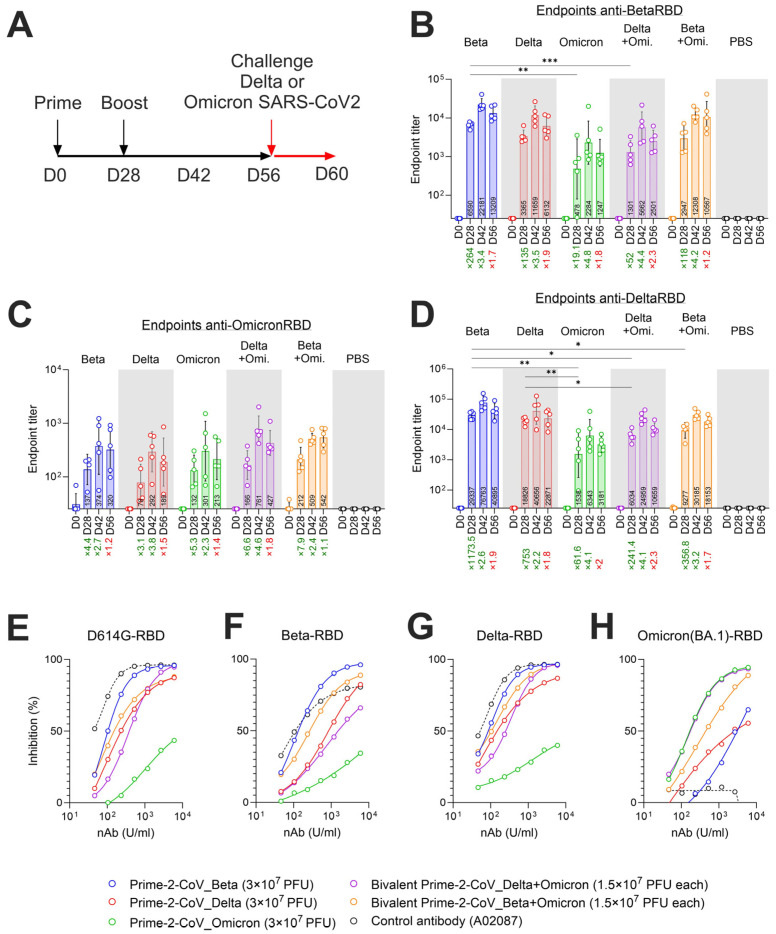
(**A**) Timeline overview of the hamster challenge study, with injections of either PBS or Prime-2-CoV vaccine at days 0 and 28, in monovalent (Beta, Delta, Omicron BA.1) or bivalent (Delta + Omicon, Beta + Omicron) injection formats. All animals were subsequently challenged with SARS-CoV-2 Delta or Omicron (BA.1) strain at day 56 and monitored for 4 days; (**B**) Anti-SARS-CoV-2 Beta RBD endpoint titer measurement via ELISA in hamster sera at indicated timepoints pre-challenge with SARS-CoV-2_Delta; (**C**) Anti-SARS-CoV-2 Omicron RBD endpoint titer measurement via ELISA in hamster sera at indicated timepoints pre-challenge with SARS-CoV-2_Delta; (**D**) Anti-SARS-CoV-2 Delta RBD endpoint titer measurement via ELISA in hamster sera at indicated timepoints pre-challenge with SARS-CoV-2_Delta; (**E**–**H**) Surrogate virus neutralization test (sVNT) of sera from vaccinated animals at day 56. Neutralizing antibodies against RBD from (**E**) the ancestral strain D614G, (**F**) Beta, (**G**) Delta or (**H**) Omicron (BA.1) were assayed. Statistical analysis was conducted using GraphPad Prism (v10.1.1). Comparisons were performed using two-way ANOVA. Symbols of statistical significance: *, *p* < 0.05; **, *p* < 0.01; ***, *p* < 0.001.

To evaluate the protective efficacy of Prime-2-CoV vaccine candidates injected with a bivalent formulation (Beta + Omicron or Delta + Omicron), we performed a challenge study on a Syrian hamster animal model. Animals were primed at day 0 with 3 × 10^7^ plaque-forming units (PFUs) of Prime-2-CoV variants (monovalent formulation) or 1.5 × 10^7^ PFUs of each variant tested (bivalent formulation) in a single injection for a bivalent formulation, mirroring potential clinical protocols. A booster vaccination, consistent with the initial formulation, was administered on day 28. Samples for serology and neutralization assays were collected at days indicated in the figure (Figure 4A). Animals were challenged intranasally with either SARS-CoV-2 Delta or Omicron strains at day 56. The progression of the disease was monitored until day 60, the final day of the study (Figure 4A).

### 3.5. Monovalent and Bivalent Prime-2-CoV Vaccinations Elicit RBD-Specific Binding and Neutralizing Antibodies in Hamsters

Priming with Prime-2-CoV_Beta in the monovalent formulation elicited high titers of anti-BetaRBD and anti-DeltaRBD-specific antibodies at day 28, showing an increase of 264-fold and 1173-fold, respectively, compared to baseline levels (day 0) (Figure 4B,D). In animals vaccinated with the bivalent formulation, Prime-2-CoV_Beta elicited lower titers of Beta RBD-specific antibodies compared to the monovalent formulation, likely due to the half dose of each construct. After the booster vaccination on day 28, the endpoint titers of Beta and Delta RBD-specific antibodies increased in all Prime-2-CoV-vaccinated animals, ranging from 2.6-fold to 4.8-fold increases, measured at day 42. Omicron RBD-specific antibodies’ endpoint levels were generally lower than those for Beta and Delta RBD-specific values across all Prime-2-CoV vaccine candidates. They elicited peak levels of antibodies at day 42, 2 weeks after the booster vaccination. These levels slightly decreased at day 56, except for the bivalent Beta + Omicron candidate, where the mean level remained constant (Figure 4C).

The strain-specific neutralizing capacity of Prime-2-CoV-elicited antibodies in hamsters was assessed via the surrogate virus neutralization test (sVNT). This assay measures the inhibition of binding between the ancestral D614G-RBD and recombinant human ACE2 protein. Sera from hamsters vaccinated with bivalent Beta exhibited the highest neutralization potency, with an EC_50_ of 64.22 U/mL, followed by the monovalent Prime-2-CoV Beta + Omicron (EC_50_ 82.54), monovalent Prime-2-CoV Delta (EC_50_ 92.14), bivalent Delta + Omicron (EC_50_ 360.60), and finally, the monovalent Prime-2-CoV Omicron (EC_50_ 1064), which had minimal neutralization potency (Figure 4E). When tested against the SARS-CoV-2 Beta RBD protein, sera from monovalent Prime-2-CoV Beta-vaccinated hamsters exhibited the highest neutralization potency, as expected, with an EC_50_ of 104.1 U/mL, followed by the bivalent Beta + Omicron (EC_50_ 256.9), the bivalent Delta + Omicron (EC_50_ 767.2), the monovalent Prime-2-CoV delta (EC_50_ 815.2) and the monovalent Prime-2-CoV Omicron (EC_50_ < 10^6^) (Figure 4F). Delta-RBD testing showed that the monovalent Prime-2-CoV Beta again exhibited the highest neutralization potential (EC_50_ 104.7), followed by the bivalent Beta + Omicron (EC_50_ 151.9). Slightly lower RBD-ACE2 inhibition was observed in wells treated with sera of monovalent Prime-2-CoV Delta-vaccinated and bivalent Omicron–Delta-vaccinated hamsters. Similarly to D614G- and Beta-RBD assays, sera from monovalent Omicron-vaccinated animals showed weak inhibition against Delta-RBD, highlighting the significant differences between the RBD of Omicron and earlier strains (Figure 4G). When tested against Omicron (BA.1)-RBD in the sVNT assay, the Omicron-containing Prime-2-CoV vaccine candidates exhibited the highest inhibition capabilities (Figure 4H). The control human antibody A02087—a standard intended for the calibration of SARS-CoV-2 neutralizing antibodies—showed very good neutralization activity against D614G- and Delta-RBD, slightly lower against Beta-RBD and a total lack of inhibition potential against Omicron (BA.1), as reported by the manufacturer.

### 3.6. Monovalent and Bivalent Prime-2-CoV Vaccine Candidates Lower Viral Loads in Hamster Airways after SARS-CoV-2 Challenge

The protective effect conferred by the vaccine candidates against SARS-CoV-2 infection was measured on days 0–4 after challenge with SARS-CoV-2 Delta or Omicron strains in a hamster model. Throat swabs were collected for the quantification of viral RNA and infectious virus quantified by RT-qPCR and TCID_50_ assays.

**Figure 5:**
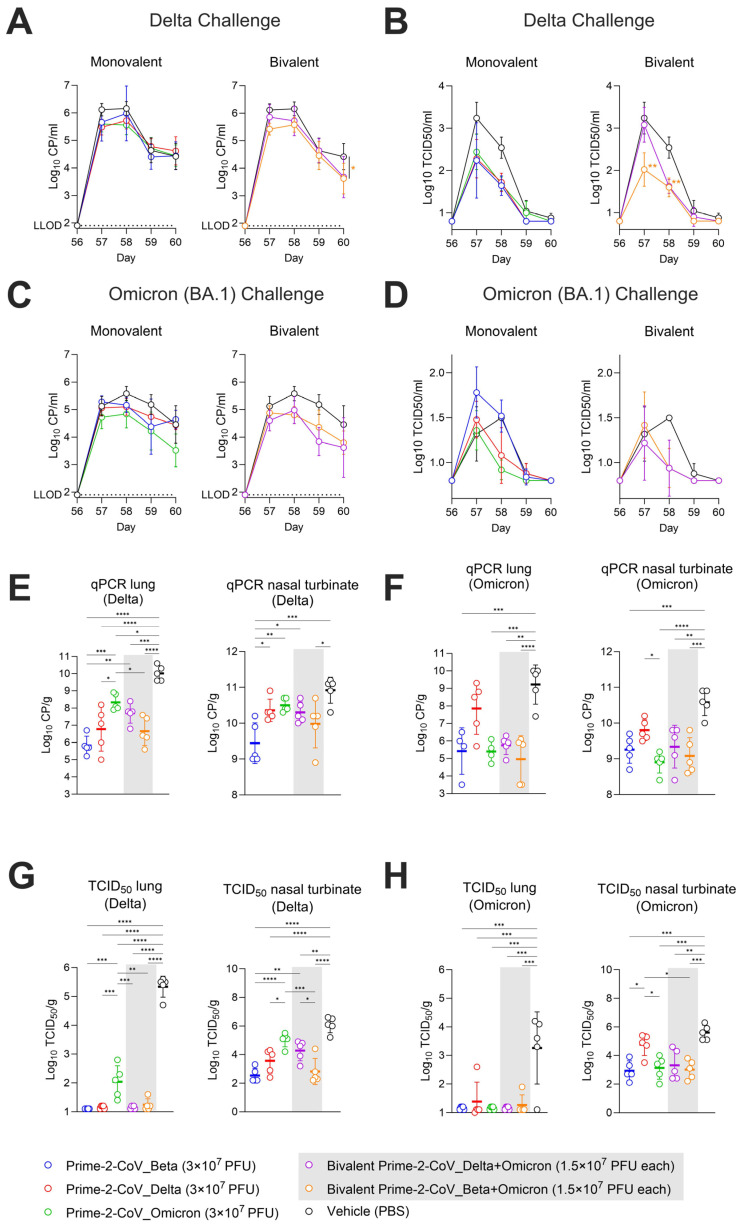
(**A**) qPCR quantification of SARS-CoV-2 viral RNA in throat swabs post-SARS-CoV-2 Delta strain challenge (d56) for monovalent (left panel) or bivalent vaccination strategies (right panel); (**B**) TCID_50_ viral load quantification in throat swabs of the same animals for monovalent (left panel) and bivalent injection strategies (right panel); (**C**) qPCR quantification of SARS-CoV-2 viral RNA in throat swabs post-SARS-CoV-2 Omicron (BA.1) strain challenge (d56) for monovalent (left panel) or bivalent vaccination strategies (right panel); (**D**) TCID_50_ viral load quantification in throat swabs of the same animals for monovalent (left panel) and bivalent injection strategies (right panel); (**E**) qPCR quantification of viral RNA in lung samples (left panel) and nasal turbinates (right panel) at day 60 for Delta-challenged animals; (**F**) qPCR quantification of viral RNA in lung samples (left panel) and nasal turbinates (right panel) at day 60 for Omicron (BA.1)-challenged animals; (**G**) TCID_50_ quantification of replicating viruses in lung samples (left panel) and nasal turbinates (right panel) at day 60 for Delta-challenged animals; (**H**) TCID_50_ quantification of replicating viruses in lung samples (left panel) and nasal turbinates (right panel) at day 60 for Omicron (BA.1)-challenged animals. Statistical analysis was conducted using GraphPad Prism (v10.1.1). Comparisons were assessed using two-way ANOVA. Symbols of statistical significance: *, *p* < 0.05; **, *p* < 0.01; ***, *p* < 0.001; ****, *p* < 0.0001.

In PBS mock-vaccinated animals challenged with SARS-CoV-2 Delta, the mean viral copies/mL values peaked at days 1 and 2 post-challenge (means of 6.12 and 6.16 Log_10_ CP/mL), dropped on day 3 (mean of 4.64 Log_10_ CP/mL) and stabilized on day 4 (mean of 4.42 Log_10_ CP/mL). All groups vaccinated with monovalent Prime-2-CoV candidates followed the same pattern, albeit with slightly lower day 1 and 2 peak mean CP/mL values (Figure 5A, left panel). In animals vaccinated with the bivalent Delta + Omicron and Beta + Omicron Prime-2-CoV, day 4 mean CP/mL values did not stabilize but rather continued to decrease to means of 3.68 and 3.62 Log_10_ CP/mL, respectively (Figure 5A, right panel).

Virus titers detected in throat swabs from animals challenged with SARS-CoV-2_Delta peaked at day 1 p.c. for the control, reaching a mean of 3.24 Log_10_ TCID_50_/_mL_. All monovalent Prime-2-CoV-vaccinated animals had lower peak values on day 1; these values further declined on days 2 and 3. The measured mean titers of monovalent Prime-2-CoV Beta and Delta had already reached the lower limit of detection (0.8 Log_10_ TCID_50_/_mL_) on day 3, while the Prime-2-CoV_Omicron group reached this threshold on day 4 (Figure 5B, left panel). Bivalent vaccination with Prime-2-CoV Delta + Omicron did not prevent virus titers from reaching levels close to the control levels of TCID_50_/_mL_ on day 1 p.c. (mean of 3.08 Log_10_ TCID_50_/mL), with levels sharply decreasing on day 3 to a value comparable to the monovalent and bivalent Beta + Omicron Prime-2-CoV groups. Vaccination with bivalent Beta + Omicron led to significantly lower mean titers on days 1 and 2 p.c. than control vaccination (Figure 5B, right panel).

In animals challenged with SARS-CoV-2 Omicron (BA.1), vaccinations with monovalent Prime-2-CoV_Omicron and both bivalents Delta + Omicron and Beta + Omicron exhibited a trend of lower-than-control levels of detected viral RNA (Figure 5C).

Virus load quantification in throat swabs of Omicron-challenged animals showed very low titers overall in comparison with those observed after the Delta challenge. Similarly to viral RNA quantification results, vaccinations with monovalent Prime-2-CoV_Omicron and both bivalents Delta + Omicron and Beta + Omicron resulted in lower mean titers at days 2 and 3 post-challenge (Figure 5D).

Viral RNA and viral titers were also quantified in the lungs and nasal turbinates of sacrificed animals on day 4 post-challenge using RT-qPCR. In control animals mock-vaccinated with PBS and challenged with the Delta strain, the detected levels reached 10 Log_10_ CP/g of tissue (Figure 5E, left panel) and 10.9 Log_10_ CP/g in nasal turbinates (Figure 5E, right panel). For all Prime-2-CoV-vaccinated groups, significantly lower CP/g values were observed in the lungs, ranging from 5.8 Log_10_ for monovalent Beta, 6.7 Log_10_ for bivalent Beta + Omicron and up to 8.3 Log_10_ for monovalent Prime-2-CoV_Omicron (Figure 5E, left panel). A similar pattern was observed in nasal turbinates, where Prime-2-CoV-vaccinated animals showed significantly lower amounts of viral RNA compared to controls. Both Beta + Omicron and Delta + Omicron bivalent-vaccinated groups had lower viral RNA amounts than the monovalent-vaccinated Omicron group in the lungs and nasal turbinates. Of note, bivalent Delta + Omicron showed a higher mean CP/g value than monovalent Delta in lungs but not in nasal turbinates, although this difference was not statistically significant (*p* = 0.4), suggesting a dose-dependent protection of Prime-2-CoV_Delta in the lungs against a Delta challenge.

When challenged with Omicron (BA.1), Prime-2-CoV vaccination led to a significant decrease in viral RNA levels in the lung tissue, with a minimum reduction of 3.5 Log_10_ across all groups, except for the monovalent Prime-2-CoV_Delta group, which showed only a 1.3 Log_10_ reduction (Figure 5F, left panel). In nasal turbinates, the best protection was conferred by Prime-2-CoV_Omicron (1.7 Log_10_ decrease vs. control) and bivalent Beta + Omicron (1.4 Log_10_ reduction vs. control) (Figure 5F, right panel).

Infectious virus amounts in the upper and lower respiratory tracts were assessed via TCID_50_ assay. Unlike RT-qPCR, which quantifies both replicating and destroyed/non-replicating viruses, the TCID_50_ assay measures only the infectious, replicating viruses, providing more precise information on vaccine efficacy.

Vaccination with the monovalent Prime-2-CoV_Beta, _Delta and the bivalent Delta + Omicron and Beta + Omicron led to barely detectable viral loads in Delta-challenged animal lungs at day 4 post-challenge. The monovalent Prime-2-CoV_Omicron-vaccinated animals showed a 2 Log_10_ TCID_50_/g viral load, corresponding to a 3.3 Log_10_ decrease, compared to control animals (5.34 Log_10_/g mean viral load) (Figure 5G, left panel). In nasal turbinates, higher viral loads were detected overall; however, significant reductions were observed particularly in the monovalent Prime-2-CoV_Beta and bivalent Beta + Omicron groups (Figure 5G, right panel).

In Omicron-challenged animals, most vaccinated animals showed viral loads at the lower limit of detection in the lungs. Notably, previous studies reported significantly lower viral loads in Omicron-challenged than in Delta-challenged hamsters [15,16]. Consistent with these results, the viral loads detected in the lungs of Omicron-challenged mock-vaccinated animals were significantly lower than those detected in Delta-challenged animals (Figure 5H, left panel). However, this difference was not observed in nasal turbinates, with TCID50/g reaching 6 Log_10_ for both Delta and Omicron challenges. Prime-2-CoV vaccination led to a significant reduction in mean TCID_50_/g in nasal turbinates by 2.6 Log_10_ for monovalent Prime-2-CoV_Beta and bivalent Beta + Omicron. Interestingly, no significant additive protective effect of Beta and Omicron was observed, the viral load in the bivalent Beta + Omicron and monovalent Beta and monovalent Omicron being similar (Figure 5H, right panel).

Although body weight loss measurements showed that Prime-2-CoV-vaccinated animals exhibited a marked recovery at days 3 and 4 post-SARS-CoV-2 challenge, indicative of the protective effect of vaccination (Supplementary Figure S2A–D), the severity and extent of alveolitis, bronchi(oli)tis, tracheitis and rhinitis in Prime-2-CoV-vaccinated animals challenged with either Delta or Omicron SARS-CoV-2 did not differ significantly compared to PBS control-vaccinated animals (Supplementary Figure S3A–F).

In summary, vaccination protected the lungs from SARS-CoV-2 infection, except in monovalent Omicron-vaccinated animals challenged with the Delta strain and Delta-vaccinated animals challenged with the Omicron strain. While not completely preventing infection in nasal turbinates, Prime-2-CoV vaccination significantly reduced the viral loads in all conditions compared to non-vaccinated controls, with the noted exceptions for monovalent Omicron-vaccinated animals challenged with the Delta strain and Delta-vaccinated animals challenged with the Omicron strain.

## 4. Discussion

In this study, we characterized the immune response to Prime-2-CoV_Beta in vitro and in mice while evaluating the mono- and bivalent formulation strategies of Prime-2-CoV candidates in Syrian hamster models. We showed that Prime-2-CoV_Beta induces effector cytokines in human PBMCs independent of the transgene expression. Although Prime-2-CoV_Beta did not boost anti-RBD antibody titers in mRNA-vaccinated, pre-immunized mice when administered at short intervals, the pre-existing immunity to spike protein did not affect the immune response to the Orf vector itself. Indeed, the antibody titers against F1L (ORF059) are low after one Prime-2-CoV_Beta vaccination as well as not neutralizing [5]. Therefore, F1L titers can be boosted with a second Prime-2-CoV_Beta vaccination. As reported previously for an approved mRNA vaccine, Prime-2-CoV_Beta induced polyfunctional CD4+ and CD8+ T cells directed against the spike protein in vaccinated mice. One potential limitation of our study lies in the exclusive use of female mice. In a hamster challenge model, we showed that Prime-2-CoV_Beta induced cross-neutralizing antibodies against D614G, Delta and Omicron BA.1 strains of SARS-CoV-2. Finally, we showed that mono- and bivalent Prime-2-CoV vaccination strategies, including Prime-2-CoV_Beta-containing formulations, induced an immune response, which mostly prevented the lungs of hamsters from carrying a viral load 4 days after SARS-CoV-2 challenge. However, this vaccination did not significantly affect the severity of the disease.

The UV inactivation of Prime-2-CoV_Beta, leading to the abrogation of protein translation, did not significantly change the murine T-cell activation profile or the human PBMC effector cytokine profile compared to non-treated Prime-2-CoV_Beta. This suggests that the expression of the transgenes has a minimal effect on the overall induction of the immune system, as the PBMCs of likely pre-immune individuals exhibited similar effector cytokine profiles for both UV and non-treated Prime-2-CoV_Beta infection. This is in line with previous studies describing the adjuvant and overall immune stimulation effect of inactivated Orf virus [17,18,19].

Prime-2-CoV_Beta administered as a third and fourth (booster) dose 2 weeks after mice were vaccinated with a primary series of two mRNA vaccines, corresponding to the time of the peak antibody response, failed to boost anti-Beta RBD endpoint titers, suggesting that there could be a ceiling or maximum antibody response for the dose tested. In the ORFEUS Phase I clinical trial investigating the safety and immunogenicity of Prime-2-CoV_Beta in mRNA-pre-vaccinated subjects, the fold increases in SARS-CoV-2 neutralizing antibody levels after Prime-2-CoV_Beta vaccination negatively correlated with pre-existing antibody levels in participants 18–55 years of age (Klinkhard U et al., manuscript under review). Similar observations were reported in human studies with homo- and heterologous COVID-19 booster vaccines [20,21,22]. While we did not directly measure for neutralizing antibodies, these results nevertheless highlight the importance of the timing of Prime-2-CoV_Beta booster injection to achieve an optimal immunization outcome.

Our Prime-2-CoV_Delta vaccine candidate was designed at the beginning of the SARS-CoV-2 Delta wave, when most of the spike sequences in publicly available databases did not yet contain the K417N mutation. Subsequently, it became evident that the Delta variant consistently exhibited this mutation. Therefore, in our hamster challenge study, there was a discrepancy between the RBD sequence in Prime-2-CoV_Delta used for vaccination (K417) and the RBD sequence of the challenge SARS-CoV-2 Delta strain virus (K417N). It is important to consider this discrepancy when interpreting our findings. This mismatch could explain why Prime-2-CoV_Beta, which has the K417N mutation, provided superior protection, as shown by the reduced viral loads in the lungs, compared to Prime-2-CoV_Delta in animals challenged with SARS-2-CoV_Delta.

Surprisingly, monovalent Prime-2-CoV_Beta and Prime-2-CoV_Omicron demonstrated a similar protective effect against Omicron challenge, with comparable levels of viral RNA and viral loads (TCID_50_) detected in the lungs and nasal turbinates and with similar weight loss profile and disease severity. As a consequence, the bivalent format (Beta + Omicron), containing a half dose of each construct, exhibited similar properties to both parental constructs, with no apparent additive effect.

The discordance between the reduction in the viral loads in the lungs of vaccinated animals and the unchanged pathology observed is interesting. Although active viral replication was mostly undetectable at day 4, the relatively small difference in peak viral loads at days 1–2 did not correspond to a decrease in disease severity in the animals.

In conclusion, self-adjuvant ORFV-based Prime-2-CoV was shown to be a promising vaccine candidate against SARS-CoV-2 infection. Overall, our findings improve the characterization of Prime-2-CoV and highlight the potential of this novel ORFV-based platform as an attractive tool for further vaccine development.

## Data Availability

The datasets presented in this article are not readily available because this is an ongoing study and due to commercial protection. Requests for access to the datasets should be directed to the corresponding author.

## Supplementary Materials

The following supporting information can be downloaded at: https://www.mdpi.com/article/10.3390/vaccines12050490/s1, Figure S1: Characterization of Prime-2-CoV_Beta (SPEk103) and UV-inactivated Prime-2-CoV_Beta (SPEk103UV30); Figure S2: Hamster body weight change after SARS-CoV-2 challenge; Figure S3: Histopathology of Prime-2-CoV vaccinated hamster at day 4 post-SARS-CoV-2 challenge.
