# Supplementary material for: Novel Multi-Antigen Orf-Virus-Derived Vaccine Elicits Protective Anti-SARS-CoV-2 Response in Monovalent and Bivalent Formats
Source: Vaccines (Basel). 2024 May 1;12(5):490. doi: 10.3390/vaccines12050490 (PMC11126134; doi:10.3390/vaccines12050490)
Supplement: Supplementary file 1 [file vaccines-12-00490-s001.zip › vaccines-2961547-supplementary.pdf]

Supplementary figures

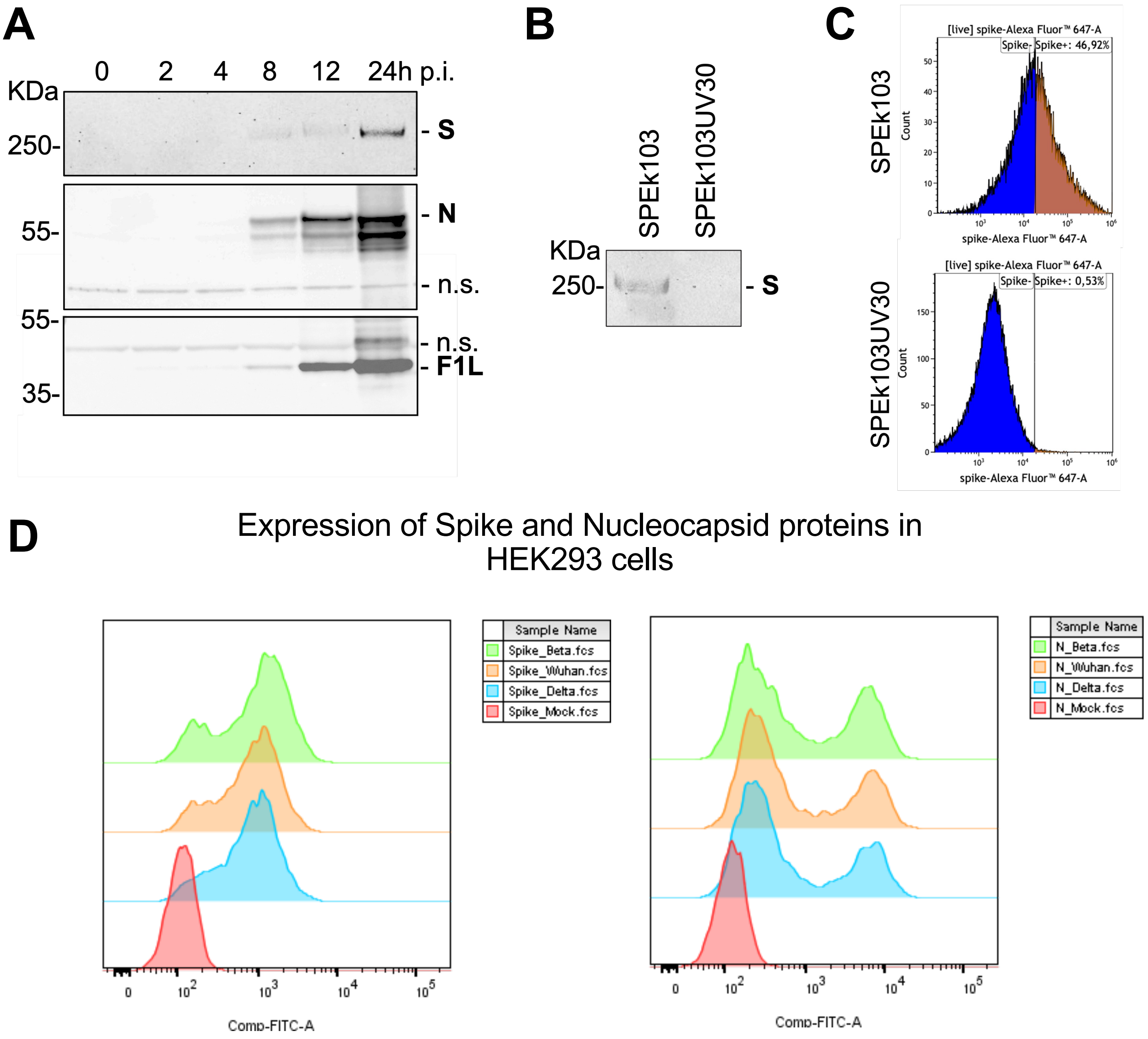

Figure S1: Characterization of Prime-2-CoV_Beta (SPEk103) and UV-inactivated Prime-2-CoV_Beta (SPEk103UV30) (A) Time-course (0-24h post-infection) of Prime-2-CoV_Beta Spike (S), Nucleocapsid (N) transgenes and Orf virus surface protein ORFV059 (F1L) expression in Vero cells infected at MOI 5 and detected by western blot. n.s. denotes non-specific labelling. B) Western blot of Prime-2-CoV_beta Spike (S) transgene expression in Vero cells infected for 24h with either Prime-2-CoV_beta (batch SPEk103) or UV-inactivated Prime-2-CoV_beta (SPEk103UV30). C) Flow cytometry assay of Vero cells infected for 48h with either Prime-2-CoV_beta (SPEk103) or UV-inactivated Prime-2-CoV_beta (SPEk103UV30) and labelled with anti-spike-Alexa 647 antibodies. D) Comparison of the expression of S and N protein in HEK cells infected with Prime-2-CoV_Beta (green), _Wuhan(D614G) (orange), _Delta (blue) and Mock infected (red).

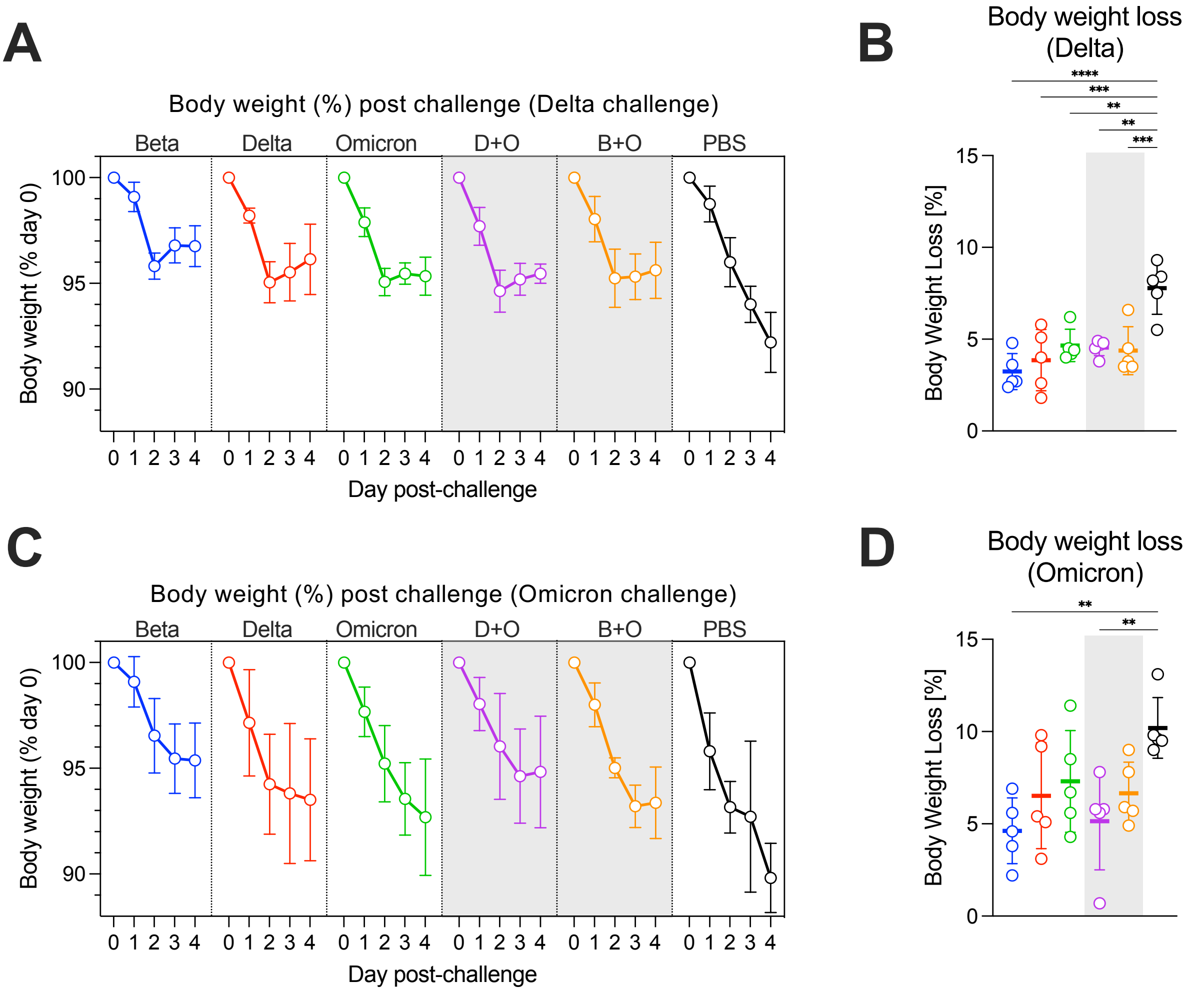

Figure S2: Hamster body weight change after SARS-CoV-2 challenge. (A) Time course of the body weight changes as percentage of day 56 (day 0 of the challenge) post-SARS-CoV-2 Delta challenge in Prime-2-CoV or PBS-vaccinated hamsters; (B) Total body weight loss on day 4 after SARS-CoV-2 Delta challenge as percentage of day 0; (C) Time course of the body weight changes as percentage of day 56 (day 0 of the challenge) post-SARS-CoV-2 Omicron(BA.1) challenge in Prime-2-CoV or PBS-vaccinated hamsters; (D) Total body weight loss on day 4 after SARS-CoV-2 Omicron(BA.1) challenge as percentage of day 0; D+O: Bivalent Prime-2-CoV_Delta+Prime-2-CoV_Omicron. B+O: Bivalent Prime-2-CoV_Delta+Prime-2-CoV_Omicron.

Supplementary text: Protection against SARS-CoV-2 challenge: body weight

The body weight of vaccinated hamsters was monitored on days 0 to 4 post-challenge. All groups showed a sharp decrease of mean body weight for the first 2 days after both Delta and Omicron challenges. A slow recovery could be observed in Delta-Challenged animals at day 3 and 4 for all vaccinated but not PBS mock-vaccinated animals (Supp. Figure 2A). In Omicron-challenged animals, this recovery was not observed. However, a stabilization of the mean body weight was observed from day 4 for Prime-2-CoV_Beta monovalent and both Delta+Omicron and Beta+Omicron bivalent vaccinated groups (Supp. Figure 2C). Mean body weight losses after 4 days, stayed under 5% for all Prime-2-CoV vaccinated groups challenged with Delta, with a lower recorder loss of 3.2% in monovalent Prime-2-CoV_Beta vaccinated group. Comparison with the 7.8% body weight loss of PBS mock-vaccinated group reached statistical difference for all groups (Supp. Figure 2B). In Omicron-challenged animals, mean body weight loss ranged from 4.6% in the Prime-2-CoV_Beta group to 7.3% in the Prime-2-CoV_Omicron group, with 10.2% in the PBS mock-vaccinated group. Bivalent Delta+Omicron and Beta+Omicron groups exhibited 5.1% and 6.7% mean body weight loss respectively (Supp. Figure 2D). Overall, mean body weight loss values were not correlated with either neutralizing or RBD-specific antibody titers.

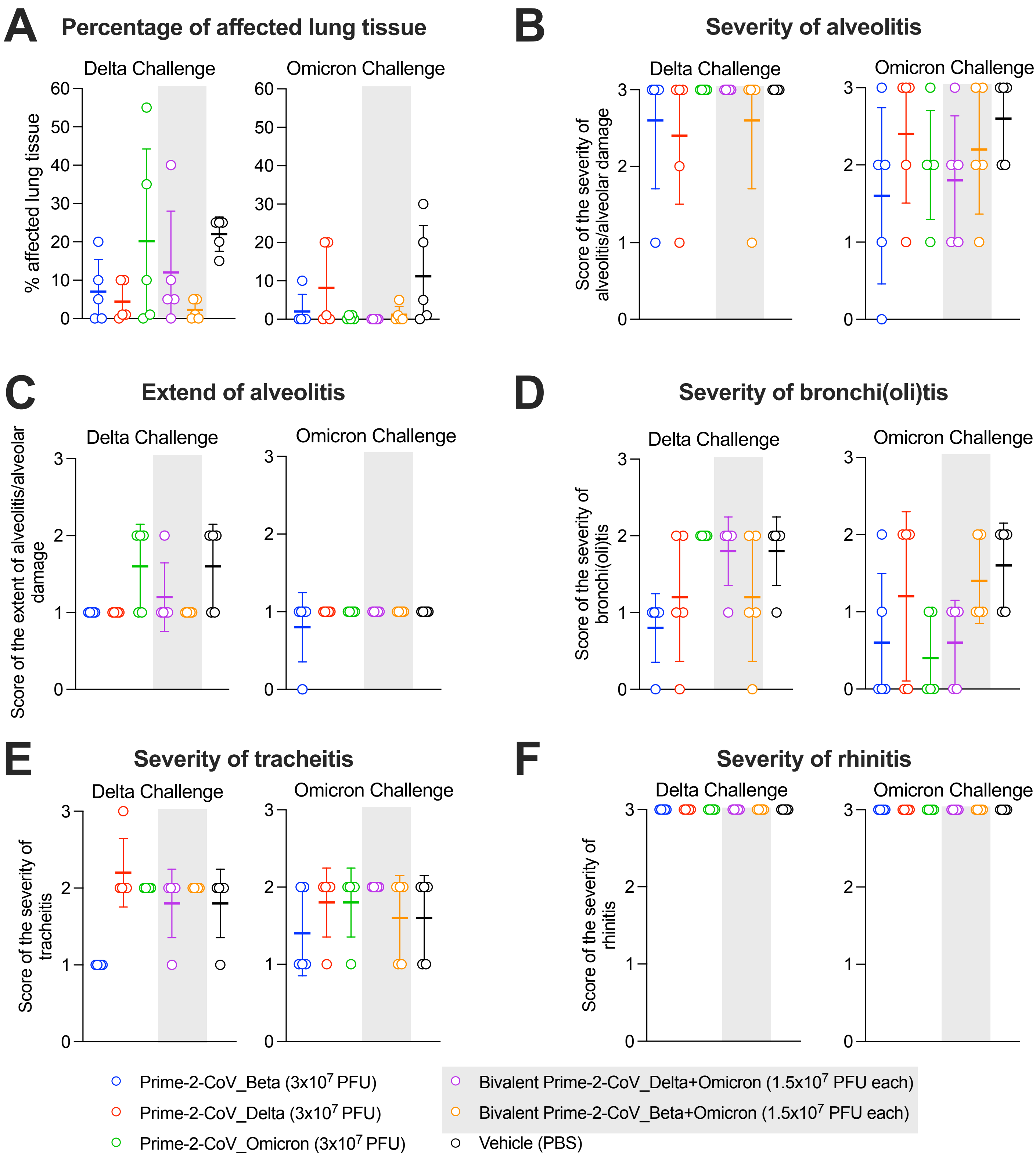

Figure S3: Histopathology of Prime-2-CoV vaccinated hamster at day 4 post-SARS-CoV-2 challenge; (A) Percentage of affected lung tissue in animals challenged with SARS-CoV-2 Delta (left panel) or Omicron(BA.1) (right panel); (B) Severity of the alveolitis in Delta (left panel) and Omicron(BA.1) (right panel) challenged animals. Scoring: 0 = no inflammatory cells, 1 = few inflammatory cells, 2 = moderate number of inflammatory cells, 3 = many inflammatory cells; (C) Extend of the alveolitis in lungs of animals challenged with SARS-CoV-2 Delta (left panel) or Omicron(BA.1) (right panel) Scoring: 0 = 0%, 1 = <25%, 2 = 25-50%, 3 = >50%; Severity of the bronchi(oli)tis (D), tracheitis (E) and rhinitis (F), in animals challenged with SARS-CoV-2 Delta (left panels) or Omicron(BA.1) (right panels) Scoring: 0 = no inflammatory cells, 1 = few inflammatory cells, 2 = moderate number of inflammatory cells, 3 = many inflammatory cells;
